# Spontaneous Perforation of Pyometra

**DOI:** 10.1155/IDOG/2006/26786

**Published:** 2006-03-29

**Authors:** Begüm Yildizhan, Esra Uyar, Alper Şişmanoğlu, Gülfem Güllüoğlu, Zehra N. Kavak

**Affiliations:** Department of Obstetrics and Gynecology, Marmara University Hospital, Faculty of Medicine, Marmara University, 81326 Haydarpaşa, Istanbul, Turkey

## Abstract

Pyometra is the accumulation of purulent material in the uterine cavity. Its reported incidence is 0.01−0.5% in gynecologic patients; however, as far as elderly patients are concerned, its incidence is 13.6% [3]. The most common cause of pyometra is malignant diseases of genital tract and the consequences of their treatment (radiotherapy). Other causes are benign tumors like leiomyoma, endometrial polyps, senile cervicitis, cervical occlusion after surgery, puerperal infections, and congenital cervical anomalies. Spontaneous rupture of the uterus is an extremely rare complication of pyometra. To our knowledge, only 21 cases of spontaneous perforation of pyometra have been reported in English literature since 1980. This paper reports an additional case of spontaneous uterine rupture.

## CASE REPORT

A 92-year-old woman with severe abdominal pain and vomiting for 24-hour duration was admitted to our hospital. Her gynecologic history was unremarkable having undergone an eventful menopause. She had no history of postmenopausal bleeding or increased vaginal discharge. On the physical examination, her abdomen was very tender, distended, and showed muscle rigidity. Rebound tenderness was absent. Bowel sounds were hypoactive. Her blood pressure was 110/65 mmHg, pulse rate was 114 beats/min, and axillary temperature was 36.9°C. Laboratory studies demonstrated a white cell count of 5100/mm^3^ with 92.3% neutrophilia and hemoglobin of 13 g/dL. A plain chest X-ray film showed free air under the diaphragm on both sides. The abdominal X-ray revealed no evidence of intestinal obstruction. Computed tomography scan of abdomen reported the presence of fluid within the abdominal cavity.

Emergency explorative laparotomy was performed under the diagnosis of perforation of the gastrointestinal tract. The investigation of the gastrointestinal tract and gallbladder failed to reveal a perforation. The uterus was found to have two perforations, approximately 1 cm in diameter each, both in the uterine fundus, and purulent material exuding from the uterine cavity was identified. The uterus was soft and slightly enlarged. Both parametriums were thickened and inflammatory changes were present. The fallopian tubes and the ovaries were normal. A total abdominal hysterectomy and bilateral salpingo-oophorectomy were performed. Culture of the pus grew *Escherichia coli* and *Bacteroides fragilis*. Histological examination revealed pyometra with no evidence of malignancy.

She was observed in the intensive care unit with strict management of respiration and circulation for postoperative three days. On the third postoperative day, she was transferred to the gynecology unit. Under the antibiotherapy with cefepime and metronidazole, her condition improved postoperatively. However, on the tenth postoperative day, wound dehiscence occurred and secondary wound closure was performed. No other complications have occurred, and as the patient completely recovered, she was discharged on the eighteenth postoperative day.

## DISCUSSION

Pyometra, or pyometrium, is defined as the accumulation of pus in the uterine cavity resulting from interference with its natural drainage. It is an uncommon condition that occurs mainly in postmenopausal women and is rare in the premenopausal age group [18]. The classic triad of symptoms in patients with pyometra consists of purulent vaginal discharge, postmenopausal bleeding, and lower abdominal pain [2]. Various malignant and benign diseases have been shown to cause pyometra [1–18].

Table 1 summarizes the 22 cases of spontaneous uterine rupture since 1980, including our case. All cases were postmenopausal elderly females, mostly in the seventh or eighth decade, except for 34- and 41-year-old women. The age at diagnosis ranged from 34 to 92 years with a mean of 75.3 years. The most common presenting symptoms were abdominal pain (95.5%), vomiting (41.0%), nausea (9.1%), and fever (9.1%). The most prevalent preoperative diagnosis was generalized peritonitis (47.4%), pneumoperitoneum (47.4%), and perforation of gastrointestinal tract (36.8%). In only 3 cases (15.8%), perforation of pyometra was suspected. Laparotomy was performed in all cases except case 12 since her general condition was poor [3]. Hysterectomy was performed in 18 cases. The location of perforation was in the fundus in 18 patients (85.7%). The bacteriological studies of intraperitoneal pus were positive in 17 cases, in one case it was negative, and in 4 cases it was not mentioned in the article. Mixed infection with both anaerobes and aerobes was detected in most of the patients. Histologically, 7 cases (35%) were associated with malignant disease, and 2 cases (10%) were associated with leiomyoma. In 10 patients, no apparent cause could be identified.

Pyometra is a rare event in general population but more common in elderly women. It is caused by impairment of natural drainage of the cervix as a result of benign or malignant diseases. A detailed pelvic examination should be performed to rule out the associated malignancies. The diagnosis of pyometra is difficult, because it is usually asymptomatic.

## CONCLUSION

Ruptured pyometra should be kept in mind in elderly women presenting with acute abdomen as an unusual but serious condition.

## Figures and Tables

**Table 1:** Cases of spontaneous perforation of pyometra reported since 1980 to date.

Case	Reference no	Year	Age	Symptoms	Provisional diag	Causative disease	Perforation site	Bacterial culture	Treatment	Outcome

1	[4]	1981	86	AP	GP, PNP	Rectum Ca	nm	*Coli* like rods, *Clostridia*	SVH + sigmoidostomy	Alive
2	[5]	1982	86	AP	GP	(—)	Fundus	nm	SVH	Died
3	[6]	1985	77	AP, N	GP	Endometrium Ca	Fundus	*E coli*, *B fragilis*	TAH + BSO	Died
4	[7]	1985	78	AP, N, V	nm	(—)	Fundus	nm	TAH + BSO	nm
5	[8]	1985	67	AP	PPU, PNP	(—)	Fundus	nm	TAH + BSO	Alive
6	[8]	1985	77	AP, V	AA	Sigmoid Ca	Fundus	nm	SVH + sigmoidectomy	Alive
7	[9]	1986	41	AP, V	PGIT, PNP	Leiomyoma	Right side	*B fragilis*	TAH	Alive
8	[10]	1989	73	AP, V, D	PGIT	(—)	Fundus	*S intermedius*	TAH	Alive
								Anaerobic *streptococci*		
9	[11]	1989	85	AP	PGIT, PNP	Leiomyoma	Fundus	*E coli*, *B fragilis*	TAH + BSO	Died
10	[12]	1991	82	AP, V	PGIT	(—)	Fundus	*E coli*, *B vulgaris*	TAH + BSO	Died
11	[2]	1993	67	AP, GB	GP, PP, PNP	Cervix Ca	Fundus	(—)	SVH + BSO	Alive
12	[3]	1995	86	AP, F	PPU	(—)	Fundus	*B fragilis*, *E coli*	Aspiration and drainage	Died
13	[13]	1996	80	AP, VD	PGIT, PNP	Endometritis	Anterior wall	*E coli*	TAH	Alive
14	[14]	1999	88	V	GP, PGIT, PNP	(—)	Fundus	*E coli*	TAH + BSO	Alive
15	[1]	2000	34	AP	GP	Cervix Ca	Left cornual region	*B fragilis*, *streptococci*	Drainage and PL	Alive
16	[1]	2000	72	AP	nm	Cervix Ca	Fundus	*B fragilis*	Drainage and PL	Died
17	[1]	2000	76	AP	AD	(—)	Fundus	*E coli*	Drainage and PL	Alive
18	[15]	2000	86	AP, F	GP, PP, PNP	Adenomyozis	Fundus	*C sphenoides*	SVH	Alive
19	[16]	2000	66	AP	nm	(—)	Fundus	*P mirabilis*, *klebsiella*	TAH + BSO	Died
20	[17]	2001	69	AP, V	GP	nm	Fundus	Anaerobes	TAH	Died
21	[17]	2001	89	AP, V	GP, PP	nm	Fundus	*E coli*	TAH + BSO	Died
22	∗	2004	92	AP, V	PGIT, PNP	(—)	Fundus	*B fragilis*, *E coli*	TAH + BSO	Alive

AP abdominal pain; N nausea; V vomiting; D diarrhea; F fever; VD vaginal discharge; GB genital bleeding; GP generalized peritonitis, PPU perforation of peptic ulcus; PGIT perforation of gastrointestinal tract; AC acute appendicitis; PP perforated pyometra; AD acute diverticulitis; Ca cancer; TAH total abdominal hysterectomy; BSO bilateral salpingo-oophorectomy; SVH supra-vaginal hysterectomy; PL peritoneal lavage; PNP pneumoperitoneum; nm not mentioned; ∗ the current case.
